# Low lean tissue mass can be a predictor of one-year survival in hemodialysis patients

**DOI:** 10.1080/0886022X.2018.1456451

**Published:** 2018-04-05

**Authors:** Aleksandra Rymarz, Julia Gibińska, Maria Zajbt, Wiesław Piechota, Stanisław Niemczyk

**Affiliations:** a Department of Internal Diseases, Nephrology and Dialysis, Military Institute of Medicine, Warsaw, Poland;; b Department of Laboratory Diagnostics, Military Institute of Medicine, Warsaw, Poland

**Keywords:** Body composition, inflammation, lean tissue mass, nutrition, survival

## Abstract

**Purpose:** Nutritional status has a significant impact on the outcomes in the dialysis population. The aim of this study was to evaluate the association between body composition and a one-year survival of hemodialysis patients.

**Methods:** Forty-eight patients with chronic kidney disease stage V treated with hemodialysis for more than three months were included. Body composition was assessed by bioimpedance spectroscopy (Body Composition Monitor, Fresenius Medical Care). Blood samples for serum creatinine, serum albumin, serum prealbumin, high sensitivity C-reactive protein (hsCRP), interleukin 6 (IL-6), insulin-like growth factor 1(IGF-1) concentrations were taken before the midweek dialysis session.

**Results:** Over the course of a one-year observation, seven patients died. We observed a significantly lower lean tissue index (LTI) (*p* = .013) and higher IL-6 (*p* = .032) and hsCRP levels (*p* = .011) among the patients who died. The remaining biochemical markers did not differ between these two groups. Kapplan–Meier analysis revealed a worse survival rate in patients with sarcopenia (lower than the 10th percentile for their age and gender) in comparison with those with normal LTI. However, it was not of statistical significance (*p* = .055). LTI inversely correlated with age and IL-6 and positively with IGF-1.

**Conclusions:** Sarcopenia defined as decreased LTI, is a relatively common condition among patients undergoing maintenance hemodialysis, it can also be associated with a lower one-year survival rate. Decreased lean tissue mass can be associated with old age, lower IGF-1 levels and higher IL-6 levels. Body composition assessment may provide prognostic data for hemodialysis patients.

## Introduction

In the general population obesity is associated with a higher mortality rate. In hemodialysis patients with increasing body mass index (BMI), the survival rate improves [1,2]. This phenomenon has also been observed in patients with heart failure, obstructive lung disease, cancer and in elderly people [3,4]. This survival paradox can be explained by the fact that an excess of fat mass is protective in the short term, though is likely to be harmful over a longer period of time. The duration of the observation period in most survival analyzes, however, does not exceed five years, while on the other hand, undernutrition is thought to be a short-term killer [5]. Therefore, contemporary studies concentrate on analyzing the influence of body composition on the survival of patients with chronic kidney disease (CKD). Some studies revealed that a reduced amount of lean body mass is associated with an increased mortality [6]. Caetano et al. [7] observed that patients, who died during the study period, had a lower lean tissue index (LTI), lower BMI as well as a lower fat tissue index (FTI). Progressive loss of muscle mass, the main component of lean mass, and muscle strength called sarcopenia has been observed in elderly people – while their lean mass reduces, the fat mass sometimes increases. This phenotype is called sarcopenic obesity which is associated with higher morbidity and mortality. The excessive amount of adipocytes produces leptin and proinflammatory cytokines which stimulate muscle degradation, enhance sarcopenia, and accelerate cardiovascular complications [8].

Similar changes in body composition are noticed among hemodialysis patients. A significant increase in fat mass along with a decrease in muscle mass was observed during the first two years of hemodialysis therapy by Marcelli et al. [9].

Decline of muscle mass is a marker of nutritional status and one of the diagnostic criteria for recognizing protein-energy wasting [10]. Muscle mass as a main compartment of protein stores, is susceptible to many pathological conditions associated with CKD. The accumulation of uremic toxins, oxidative stress, nonspecific inflammation, insulin resistance, metabolic acidosis, vitamin D deficiency, and protein-restricted diet can induce and enhance sarcopenia. Physical inactivity is not only a consequence of these conditions but additionally accelerates the decrease in muscle mass [11].

Aside from the body composition, CKD itself is a chronic inflammation. Even in the early stages of CKD elevated levels of proinflammatory cytokines (IL-1β, IL-6, TNF-α) are observed [12]. Uremic state increases the production of these cytokines in peripheral cells and delays their removal. Chronic inflammation is proved to be a predictive factor of all-cause and cardiovascular mortality in hemodialysis patients [13].

The aim of our study was to evaluate the association between body composition measured by bioimpedance spectroscopy (BIS), and a one-year survival of hemodialysis patients.

## Methods

### Design

We performed a prospective longitudinal observational study of hemodialysis patients who met the inclusion criteria and we analyzed body composition in relation to mortality. Furthermore, biochemical parameters of protein-energy wasting and inflammatory markers were evaluated.

### Patients

Forty-eight patients with CKD stage V treated with hemodialysis for more than three months were included. Patients were recruited from a single dialysis center between September 2011 and December 2012. Individuals with end stage heart failure, chronic pulmonary disease, current malignancy, cirrhosis, clinical signs of infection, and metal parts in the body were excluded. The local ethical committee accepted the study protocol. Institutional review board (IRB) approval nr 62/WIM/2011 was obtained 2011-08–17. Each participant signed their informed consent.

### Variables collected

BIS measurements were performed using a Body Composition Monitor (Fresenius Medical Care) before the midweek dialysis session, in supine position after a 5-min rest. Electrodes were placed on the hand and foot contralateral to the arteriovenous fistula. The data obtained from BIS measurements were as follows: LTI defined as a quotient of lean mass and squared of height, LTI difference to reference (ΔLTI) defined as the difference between a patient’s lean mass and the 10th percentile for their age and gender, FTI defined as a quotient of fat mass and squared of height, FTI difference to reference (ΔFTI) defined as the difference between a patient’s fat mass and the reference range for age and gender and overhydration (OH). Sarcopenic patients were defined as those who had LTI lower than the 10th percentile for their age and gender (minus values of ΔLTI). The reference values of LTI were defined as values between 10th percentile and 90th percentile for age and gender. The reference values of FTI were defined as values between 10th percentile and 90th percentile for age and gender. For overhydration reference range was −1 to 1 L. Patients were asked to avoid the consumption of alcoholic beverages, physical exertion, visits to the sauna on the day prior to and the day of the BIS examination and to avoid eating and drinking two hours before the BIS examination. Handgrip strength (HGS) was measured using a Saehan hydraulic dynamometer. During examination, the patient remained seated with their elbow flexed at 90° and forearm in a neutral position. In hemodialysis patients measurements were performed on the arm without arteriovenous fistula, in the other patients on the non-dominant arm.

Blood samples were collected before the midweek dialysis session. The plasma was separated within 30 min, samples for measuring interleukin 6 (IL-6) and insulin-like growth factor 1 (IGF-1) levels were kept frozen at −80 °C. Concentrations of high sensitivity C-reactive protein (hsCRP) were performed by nephelometry assay (BNII Siemens) with a cutoff point of 0,8 mg/dl. IL-6 concentrations were measured using solid-phase ELISA (Quantikine HS ELISA, R&D systems, Abingdon, UK). Insulin-like growth factor 1 was measured by ELISA (DiAsource ImmunoAssays S.A, Louvain-la-Neuve, Belgium). Serum creatinine concentrations were measured using the Jaffe method (Gen.2, Roche Diagnostics GmbH, Switzerland), serum albumin levels were measured using BCP Albumin Assay Kit (Roche Diagnostics GmbH, Switzerland), serum prealbumin levels were measured using a nephelometric technology (BN II Siemens Healthineers, Germany) at the local Department of Laboratory Diagnostics.

### Statistical analysis

Statistical analysis was performed using the Statistica12 package StatSoft Poland. Categorical variables were summarized through the calculation of frequency. Continuous variables were summarized using descriptive statistics (mean, standard deviation, median and range). To check normality distribution of variables Kolomogorow–Smirnow test was used. The statistical analysis was conducted using the Mann–Whitney test in order to compare the results between the two groups. The correlation between LTI, FTI and age, creatinine, IGF-1, IL-6, albumin, prealbumin, and time of dialysis was assessed using Spearman’s rank correlation coefficient. Overall survival was calculated and survival curves were plotted using the Kapplan–Meier method, differences between groups were compared using log-rank tests. The significance of survival variables (age, sex, LTI, FTI, albumin, prealbumin, IL-6, IGF-1 levels, duration of hemodialysis, and overhydration) was evaluated using a multivariate Cox proportional hazards analysis. The results were assumed as statistically significant if *p* < .05.

## Results

Forty-eight hemodialysis patients were included. The mean age of the study population was 59.8 ± 15.5 years. 32 patients (66.7%) were men. The mean BMI was 25.2 ± 5.0 kg/m^2^, the mean LTI was 12.0 ± 2.4 kg/m^2^, the mean FTI was 12.6 ± 6.0 kg/m^2^, and the mean HGS was 21.7 ± 11.4 kg.

Twenty-seven patients (56.25%) had LTI lower than the 10th percentile for their age and gender (sarcopenic patients). Eleven patients (22.91%) had FTI above the upper limit, only 2 patients (4.16%) had FTI lower than the reference range. The rest of the group had FTI within the reference range. Among patients with FTI above the normal range (11 patients), almost all of them (10 patients) had LTI lower than normal which means that 20.83% of the study population presented a phenotype of sarcopenic obesity.

All demographic data are presented in Table 1.

**Table 1.** Demographic and clinical characteristics of the subjects (*n =* 48).

	Mean ± SD
Age (yrs)	59.83** **±** **15.54
Men (%)	66.66
Primary renal disease (%)	
PGN	18.72
Nephrosclerosis	12.50
PKD	14.64
Diabetic nephropathy	10.42
Hypertensive nephropathy	4.13
Unknown	18.71
Urological causes	12.56
Others	8.31
Duration of hemodialysis (months)	24.95** **±** **27.74
Kt/V	1.19** **±** **0.25
RRF (L)	0.9** **±** **0.7
BMI (kg/m^2^)	25.22** **±** **5.08
LTI (kg/m^2^)	12.00** **±** **2.42
FTI (kg/m^2^)	12.60** **±** **6.06
OH (L)	1.39** **±** **1.65
BCM (kg)	17.89** **±** **5.71
Serum creatinine (mg/dl)	8.56** **±** **2.81
Serum albumin (g/dl)	4.00** **±** **0.47
Serum prealbumin (mg/dl)	33.57** **±** **10.90
IGF-1 (pg/ml)	84.09** **±** **63.11
CRP (mg/dl)	1.16** **±** **1.34
IL-6 (pg/ml)	10.20** **±** **11.31

BCM: body cell mass; BMI: body mass index; CRP: C-reactive protein; FTI: fat tissue index; Kt/V: a measure of dialysis adequacy, K-dialyser clearance of urea, t-dialysis time, V-volume of urea distribution; IGF-1: insulin-like growth factor 1; IL-6: interleukin 6; LTI: lean tissue index; OH: overhydration; PKD: polycystic kidney disease; PGN: primary glomerulonephritis; RRF: residual renal function; SD: standard deviation.

Others: Alport’s syndrome, chronic hyperuricemic nephropathy, lupus nephritis, monoclonal gammapathy of undetermined significance.

**Figure 1.**
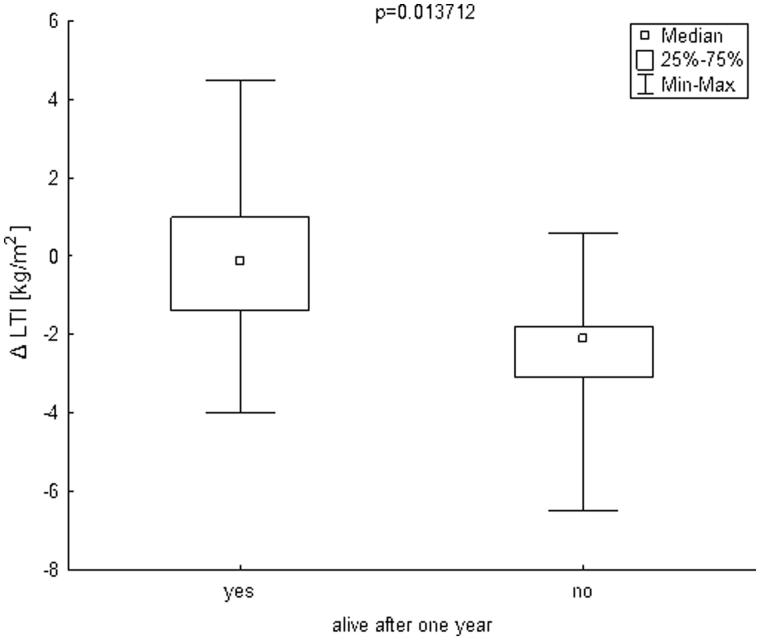
Box plot charts represent ΔLTI [kg/m^2^] in patients, who stay alive after one year (yes) and in patients, who died during one year (no). ΔLTI [kg/m^2^] – difference between patient’s lean mass and the 10th percentile for their age and gender. LTI: lean tissue index.

**Figure 2.**
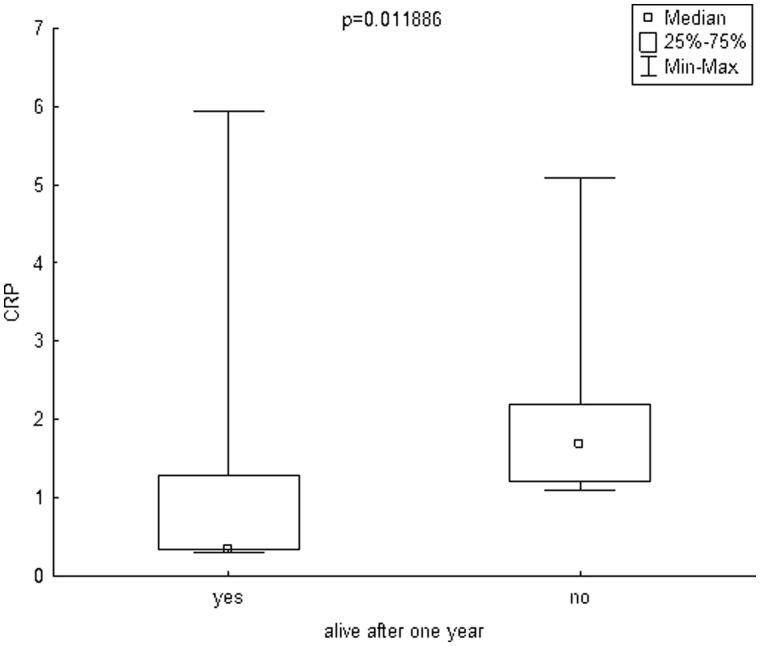
Box plot charts represent CRP [mg/dl] levels in patients, who stay alive after one year (yes) and in patients, who died during one year (no). CRP: C-reactive protein.

**Figure 3.**
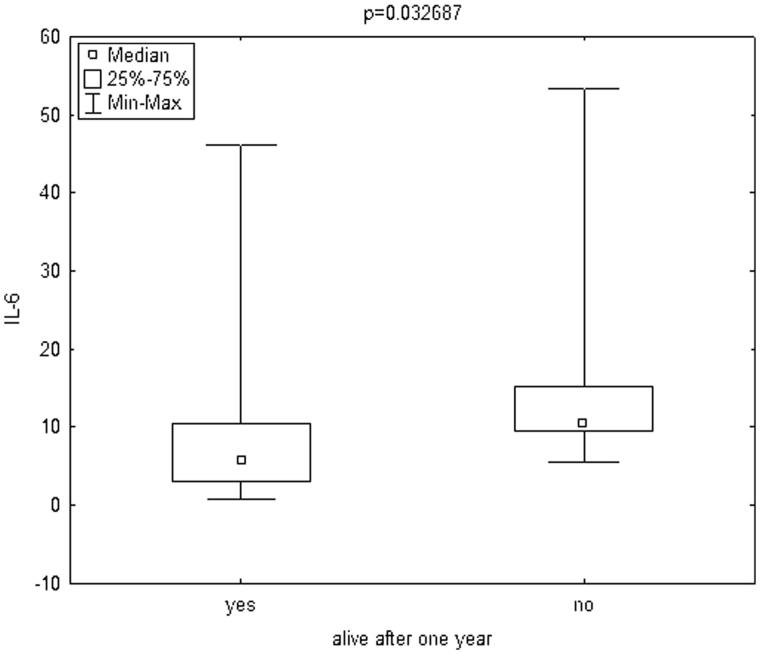
Box plot charts represent IL-6 [pg/ml] levels in patients, who stay alive after one year (yes) and in patients, who died during one year (no). IL-6: interleukin 6.

The mean follow-up was 29.93 ± 20.09 months. During one year of follow-up, 7 patients (14.58%) died. Infection was a cause of death in four cases and cardiovascular diseases in three cases. The mean age of patients who died was 64.42 ± 9.08 years. To compare the patients who died during one year of follow-up to those who survived, the Mann–Whitney test was used. Patients who died during one year of follow-up had significantly lower delta LTI (defined as the difference between a patient’s lean mass and the 10th percentile for their age and gender, Figure 1) and significantly higher hsCRP (Figure 2) and IL-6 (Figure 3). The remaining parameters studied did not differ between these two groups.

**Figure 4.**
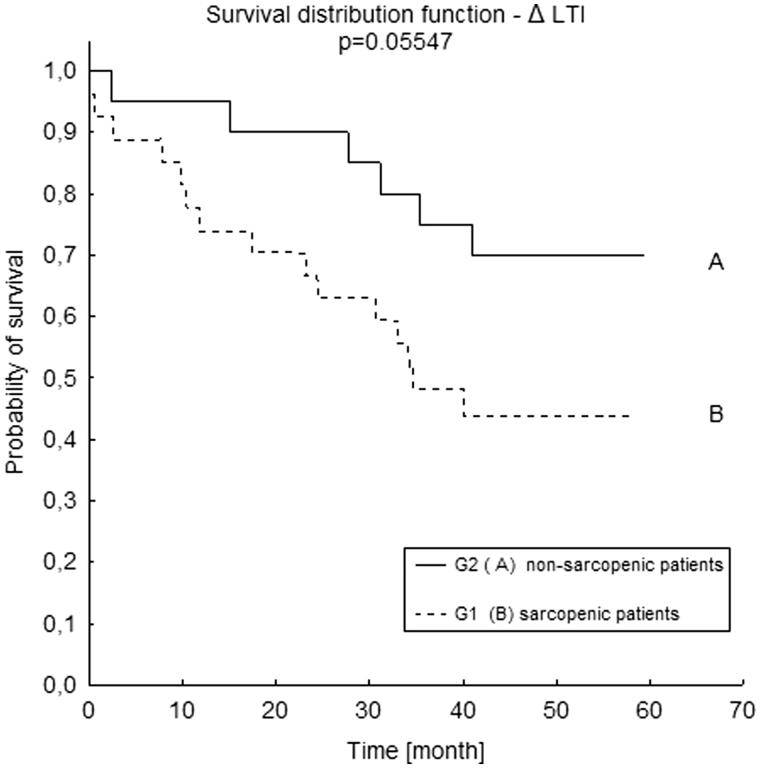
Survival distribution function according to ΔLTI in Kapplan–Meier analysis. (A) ΔLTI >0 (ΔLTI – difference between patient’s LTI and the 10th percentile for their age and gender); (B) ΔLTI <0 (ΔLTI – difference between patient’s LTI and the 10th percentile for their age and gender). LTI: lean tissue index.

In the study, we observed a lower survival rate in patients with sarcopenia (LTI lower than the 10th percentile for their age and gender), in comparison with those with normal LTI. However, it was not of statistical significance (*p =* .055). Kapplan–Meier curves are presented in Figure 4. In sarcopenic patients, the mean LTI was 11.1 ± 2.17 kg/m^2^, the mean FTI was 13.7 ± 6.40 kg/m^2^, the mean hsCRP was 1.37 ± 1.47 mg/dl, and the mean IL-6 was 9.7 ± 10.55 pg/ml. In non sarcopenic patients, the mean LTI was 13.5 ± 1.97 kg/m^2^, the mean FTI was 10.5 ± 4.97 kg/m^2^, the mean hsCRP was 0.87 ± 1.12 mg/dl, and the mean IL-6 was 10.3 ± 12.21 pg/ml.

**Figure 5.**
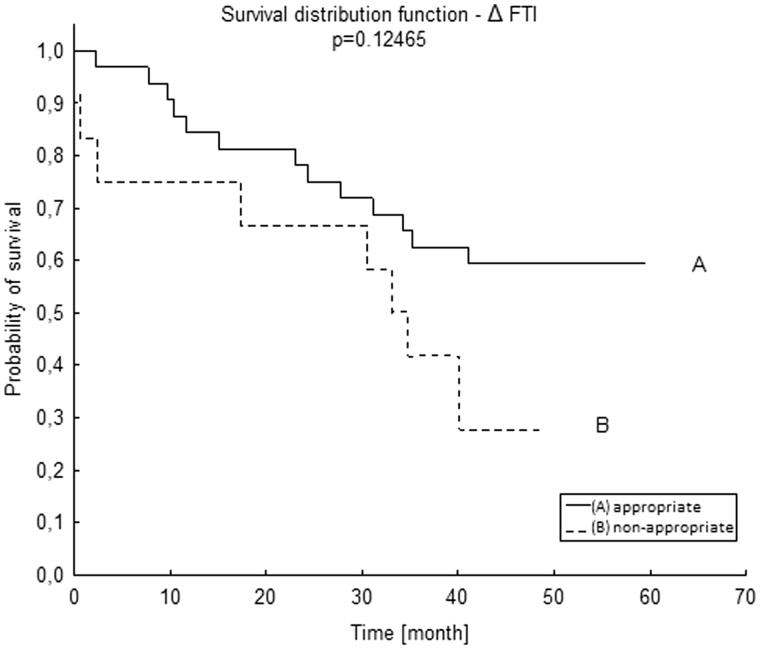
Survival distribution function according to ΔFTI in Kapplan–Meier analysis. ΔFTI – difference between patient’s FTI and the reference range for age and gender. FTI: fat tissue index.

To analyze FTI, due to the small amount of patients with FTI below the lower limit (two patients), we decided to take into account all patients with abnormal FTI together – i.e., patients with FTI above the upper limit and those who fell below the lower reference range. Patients with abnormal FTI had a worse survival than those with normal FTI, but it did not reach statistical significance (*p =* .124, Figure 5).

Spearman’s rank correlation coefficient was used to assess the association between body composition (LTI and FTI) and nutrition markers. Significant positive correlations were observed between LTI and HGS (*r* = 0.571; *p =* .001), serum creatinine concentration (*r* = 0.357; *p =* .049), IGF-1 (*r* = 0.326; *p =* .049). Statistically significant negative correlations were observed between LTI and age (*r* = −0.488; *p =* .001) and IL-6 (*r*= −0.295; *p =* .049). FTI significantly and positively correlated with age (*r* = 0.446; *p =* .001) and IL-6 (*r* = 0.357; *p =* .008). All correlation coefficient and *p* values are summarized in Table 2.

**Table 2.** The association of LTI and FTI with anthropometric measurements, biochemical markers, and some demographic data.

	LTI *r*	*p*	FTI *r*	*p*
Age	**−0.488**	.001	**0.446**	.001
HGS	**0.571**	.001	**−**0.290	>.05
Serum creatinine (mg/dl)	**0.357**	.049	**−**0.255	>.05
Serum albumin (g/dl)	**−**0.061	>.05	0.088	>.05
Serum prealbumin (mg/dl)	0.173	>.05	**−**0.010	>.05
IGF-1 (pg/ml)	**0.326**	.049	0.125	>.05
CRP (mg/dl)	**−**0.200	>.05	**−**0.168	>.05
IL-6 (pg/ml)	**−0.295**	.048	**0.357**	.008
Duration of hemodialysis (months)	**−**0.141	>.05	0.027	>.05
OH (L)	**−**0.033	>.05	**−**0.165	>.05

CRP: C-reactive protein; FTI: fat tissue index; HGS: handgrip strength; IGF-1: insulin-like growth factor 1; IL-6: interleukin 6; LTI: lean tissue index; OH: overhydration; r: correlation coefficient.

Bold font indicates statistically significant correlation.

## Discussion

The main finding of the study was that decreased LTI was associated with a worse survival rate of patients treated with hemodialysis. In our study, patients who had LTI lower than the 10th percentile for their age and gender presented a worse survival in comparison with those who had normal LTI. However, in the Kapplan–Meier analysis it was not of statistical significance (*p* = .055), most likely due to the small sample size. Furthermore, when taking into account the LTI of patients who died during one year of observation, it was significantly lower (*p =* .032) when compared to the patients who were still alive. These results are similar to those obtained by other researchers who also used BIS as a method of body composition assessment (BCM Fresenius medical Care). Caetano et al. [7] observed a worse one-year survival rate in patients with lower LTI. Marcelli et al. [14] demonstrated a higher mortality in patients with LTI lower than a 10th percentile. Rosenberger et al. [15] observed that malnutrition, when defined as a decreased LTI, was an independent predictor of mortality.

BIS assesses body composition in a three compartment model. Aside from data on lean tissue and fat mass it also provides information on the amount of overhydration. Fluid overload, which is often considerable in dialysis patients, may significantly influence the accuracy of methods based on a two compartment model [16]. Nevertheless, researchers who used other muscle mass assessment techniques also observed a worse survival rate in patients who presented a reduction in muscle mass. Ren et al. [17], who used bioimpedance analysis for body composition assessment, noticed increased mortality among sarcopenic hemodialysis patients in comparison with those with normal muscle mass. Su et al. [18] reported higher all-cause mortality in hemodialysis patients whose mid-arm muscle circumference decreased during 2.5 years of follow-up. This trend was the most significant among patients with BMI lower than 25 kg/m^2^. Moreover, the researchers who performed dual-energy X-ray absorptiometry (DEXA) for muscle mass assessment observed increased mortality among patients with reduced muscle mass [6]. The DEXA technique was once considered the reference method for body composition assessment; however, it has some limitations such as X-ray radiation, its inability to be used at the patient’s bedside and the influence of overhydration on the results [19]. In fact, when considering the diagnosis of protein-energy wasting, none of the aforementioned techniques can be recommended as the absolute ‘gold standard’ [10]. The results of our study confirm the observations of other researchers that decreased lean tissue mass is associated with worse survival.

Discussing the influence of sarcopenia on survival it seems reasonable to consider its definition. The term sarcopenia, derived from Greek, is used to describe muscle mass reduction with decreased muscle strength or simply, to describe a decreased muscle mass in the elderly [20]. These two definitions cause substantial discrepancies between the prevalence of sarcopenia presented in different publications. In our study, we observed a relatively high percentage (56.25%) of patients with sarcopenia when defined as LTI lower than the 10th percentile for their respective age and gender. Kim et al. [21] reported 33.1% of patients with sarcopenia defined as decreased LTI and reduced muscle strength, and 47.2% of patients with sarcopenia defined as only reduced LTI.

The gradual reduction of muscle mass is a process associated with age, and observed in the population without kidney insufficiency. Normally, the peak level of muscle mass is seen between the third and fourth decade of life before its subsequent steady decline is generally observed [22]. In patients with CKD, age-related muscle loss is deepened by metabolic disorders associated with renal failure such as inadequate nutrient intake, loss of nutrients, catabolic illnesses, acidemia, inflammation, low levels of or resistance to anabolic hormones like insulin, growth hormone, and IGF-1 [20]. As is generally the case among all people, muscle decline is associated with age. However, among patients with CKD, this decline is more intense than among subjects with a normal renal function [23,24]. The results of our study also correspond with these observations. There was an inverse correlation between LTI and age (*r*= −0.488, *p* = .001), whereas there was a positive correlation between lower IGF-1 levels and lower lean tissue mass (*r* = 0.326, *p* = .049).

It has been proved that CKD is associated with low-grade chronic inflammation expressed by elevated levels of cytokines (IL-6, Il-1β, TNF-α) as well as acute-phase proteins (CRP, albumin, fetuin-A) [25,26]. The association between IL-6 levels and mortality has been reported either in general population or in CKD patients [27]. The results of our study also correspond with these observations. Among the patients who died during one year of follow-up, IL-6 and hsCRP levels were higher than in the rest of the group. The most probable and obvious mechanism by which inflammation influences mortality is the involvement in cardiovascular pathologies such as atherosclerosis. IL-6 mRNA has been detected in coronary plaques and elevated IL-6 levels can predict worse outcomes in patients with ST–elevation acute myocardial infarction [28]. On the other hand, chronic inflammation is one of the factors inducing protein-energy wasting which is a predictor of increased mortality in patients with CKD. A state of inflammation stimulates catabolic processes and causes muscle degradation through the activation of ubiquitine-proteasome system and insulin resistance. As evidence of this thesis, an inverse correlation between muscle mass and inflammatory markers has been observed by some researchers [29]. Supporting this trend, our study also revealed a negative correlation between IL-6 concentration and LTI (*r*= −0.295; *p* = .049). At the same time, the IL-6 level positively correlated with FTI (*r* = 0.357; *p* = .008). A higher amount of fat mass produces an excess of proinflammatory cytokines which enhance the chronic inflammatory state. A reduced amount of lean tissue mass together with an increase in fat mass form a phenotype of ‘sarcopenic obesity’. A combination of these two pathologies associated with metabolic disorders leads to a higher risk of mortality than when each pathology occurs separately. Recent studies revealed a higher risk of death in the population of older men with sarcopenic obesity phenotype [30]. In older women, sarcopenia was proved to be a risk factor of increased mortality independently of obesity [31]. In our study almost all individuals with an excess of fat mass, also had decreased muscle mass (10 out of 11). Taking into account the entire studied group, 20% of patients presented sarcopenic obesity.

In conclusion, sarcopenia defined as decreased LTI, is a relatively common condition among patients undergoing maintenance hemodialysis, it can also be associated with a lower one-year survival rate. Decreased lean tissue mass can be associated with old age, lower IGF-1 levels and higher IL-6 levels. Body composition assessment may provide prognostic data for hemodialysis patients.

## Ethical approval

All procedures performed in studies involving human participants were in accordance with the ethical standards of the institutional research committee and with the 1964 Helsinki declaration and its later amendments or comparable ethical standards.

This article does not contain any studies with animals performed by any of the authors.
